# A case of gas in the stomach wall and portal vein following endoscopic ultrasound-guided hepaticogastrostomy

**DOI:** 10.1055/a-2848-4006

**Published:** 2026-04-20

**Authors:** Koh Kitagawa, Jun-ichi Hanatani, Shohei Asada, Yui Osaki, Tomihiro Iwata, Hiroki Kinoshita, Hitoshi Yoshiji

**Affiliations:** 1 12967 Department of Gastroenterology, Nara Medical University, Nara, Japan

**Fig. 1:**
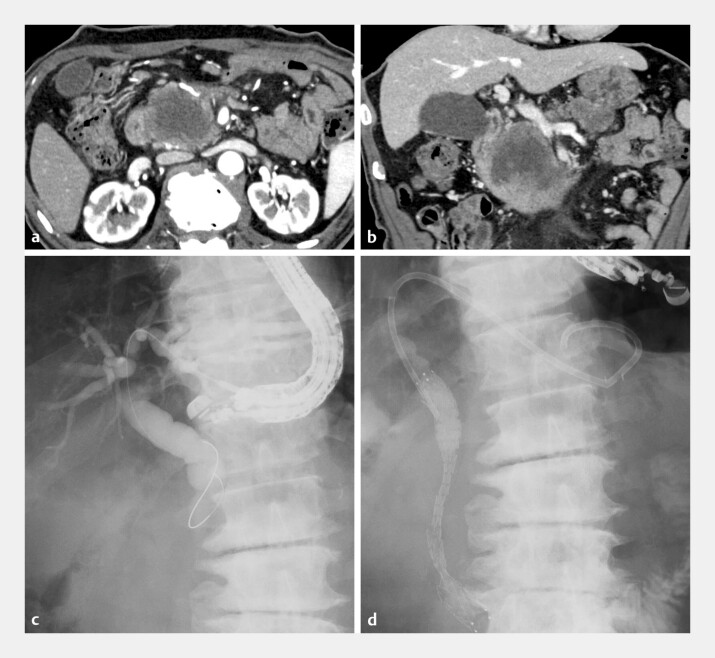
A case of obstructive jaundice caused by advanced pancreatic head cancer. **a** and **b** Abdominal contrast-enhanced computed tomography (arterial phase) revealed pancreatic head cancer with duodenal inavsion. **c** and **d** Fluoroscopic images during the initial endoscopic ultrasound-guided hepaticogastrostomy with antegarde stenting. A laser-cut metallic stent (length, 10 cm; diameter, 10 mm) was placed antegrade across the distal bile duct stricture. Subsequently, a dedicated single-pigtail plastic stent (length, 15 cm; diameter, 7 Fr) was deployed as the hepaticogastrostomy stent, with no immediate adverse events. The total procedure duration was 21 minutes.

**Fig. 2:**
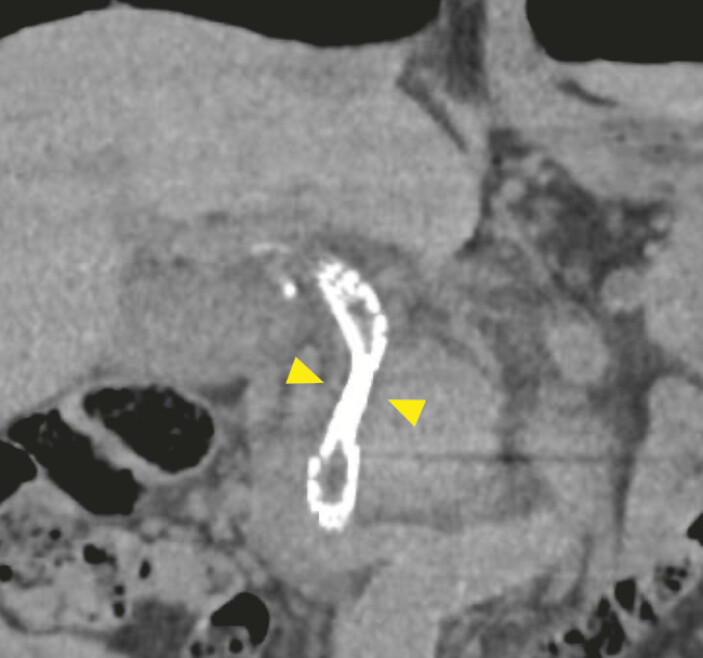
Abdominal computed tomography performed 16 days after endoscopic ultrasound-guided biliary drainage demonstrated the incomplete expansion of the antegrade metallic stent (yellow arrowheads).

**Fig. 3:**
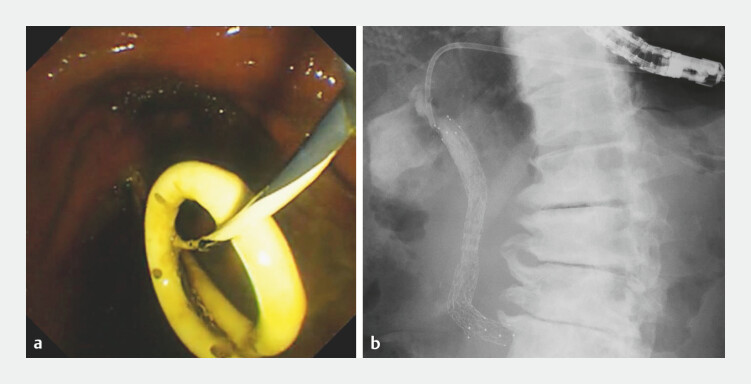
Endoscopic reintervention was performed 16 days after the initial endoscopic ultrasound-guided hepaticogastrostomy (EUS-HGS). **a** An endoscopic view showing the insertion of a 0.025-inch guidewire alongside the existing plastic stent in the HGS tract, followed by the removal of the plastic stent. **b** After balloon dilation of the biliary stricture, a new metallic stent was deployed, followed by the placement of a new plastic stent along the HGS tract. HGS, hepaticogastrostomy.

**Fig. 4:**
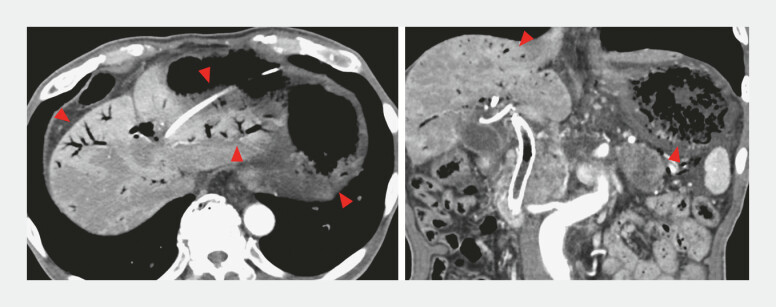
Contrast-enhanced computed tomography (arterial phase) at emergency readmission revealed extensive gas accumulation within the gastric wall and portal venous system (red arrowheads). Vital signs were stable, and there were no peritoneal irritation signs. Laboratory tests showed a normal white blood cell count, a mildly elevated C-reactive protein level of 3.01 mg/dL, and total bilirubin of 2.8 mg/dL. Acute cholangitis was ruled out based on imaging and laboratory results, and biliary drainage was considered adequate.

**Fig. 5:**
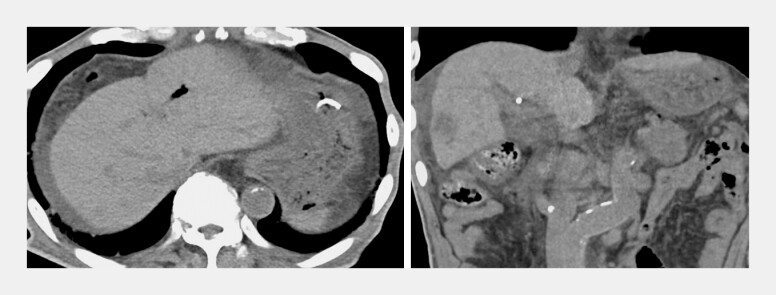
A follow-up abdominal computed tomography scan performed the day after readmission showed the spontaneous resolution of gas within the gastric wall and portal vein.

A 76-year-old man presented with obstructive jaundice caused by advanced pancreatic head cancer ( Fig. 1 **a** and **b** ). We performed endoscopic ultrasound-guided hepaticogastrostomy (EUS-HGS) with antegrade stenting ( Video 1 , Fig. 1 **c** and **d** ). However, post-discharge blood tests revealed worsening jaundice. Abdominal computed tomography (CT) suggested the incomplete expansion of the antegrade metallic stent ( Fig. 2 ). Endoscopic reintervention was performed 16 days after the initial EUS-HGS ( Fig. 3 **a** and **b** ). A guidewire was inserted alongside the existing plastic stent (PS) positioned in the HGS route, after which the PS was removed. A new metallic stent was deployed, followed by the placement of a new PS along the HGS tract. The patient was discharged 5 days after the second procedure. Ten days later, the patient experienced sudden vomiting and abdominal pain. The patient was diagnosed with emphysematous gastritis ( Fig. 4 , 1 ). Treatment consisted of fasting, intravenous fluids, and antibiotics. A follow-up abdominal CT scan performed the day after admission showed the spontaneous resolution of the gas within the gastric wall and portal vein ( Fig. 5 ). The patient resumed oral intake 4 days after diagnosis and was discharged 7 days thereafter.

A case of gas in the stomach wall and portal vein following endoscopic ultrasound-guided hepaticogastrostomy.Video 1

In this case, vomiting from duodenal stenosis, combined with gastric wall trauma during PS replacement, likely precipitated emphysematous gastritis. Notably, early endoscopic reintervention after the initial EUS-HGS, performed while the hepaticogastric anastomosis was still immature, may have further contributed to mucosal injury. Emphysematous gastritis has been reported following endoscopic procedures such as percutaneous endoscopic gastrostomy 2 and EUS-guided tissue acquisition 3 . To our knowledge, this is the first reported case associated with EUS-HGS. Endoscopists should be aware that emphysematous gastritis can occur after procedures involving the placement of stents or tubes through the gastric wall. Furthermore, it is recommended that duodenal stenosis be relieved before performing EUS-HGS, for example, by the placement of a duodenal stent.

Endoscopy_UCTN_Code_CPL_1AL_2AD
